# Metabolic adaptations of starving purple sea urchins (*Strongylocentrotus purpuratus*) surviving in the barrens

**DOI:** 10.1038/s41598-026-53701-2

**Published:** 2026-07-30

**Authors:** Leonie Venter, Du Toit Loots, Andrea C. Alfaro, Daniel S. Swezey, Daniel K. Okamoto, Maya J. Munstermann, Laura Rogers-Bennett

**Affiliations:** 1 Biomedical and Molecular Metabolism Research, Faculty of Natural and Agricultural Science, North-West University, Private Bag 1290, Potchefstroom, 2520 South Africa; 2 Department of Animal Science, Coastal and Marine Sciences Institute, University of California Davis, Davis, CA 95616 USA; 3 Bodega Marine Laboratory, Coastal and Marine Sciences Institute, University of California Davis, 2099 Westside Rd., Bodega Bay, CA 94923 USA; 4 Kashia Band of Pomo Indians of the Stewarts Point Rancheria, 1440 Guerneville Road, Santa Rosa, CA 95403 USA; 5 Department of Integrative Biology, University of California Berkeley, Berkeley, CA 94720 USA

**Keywords:** Carbohydrate metabolism, Gas chromatography-mass spectrometry, Kelp, Metabolomics, Sea urchin starvation, Biochemistry, Ecology, Ecology, Ocean sciences, Physiology, Zoology

## Abstract

Sea urchin grazing can drive kelp loss creating low productivity “barrens” with reduced biodiversity that may persist for decades. In these habitats, limited food driven by sea urchin herbivory reciprocally affects sea urchin physiology, yet the metabolic pathways enabling survival in the starvation conditions remain poorly understood. This study employed gas chromatography–mass spectrometry metabolomics to compare gonad metabolite profiles of purple sea urchins, *Strongylocentrotus purpuratus*, from barrens and adjacent kelp forest margins. Gonads from barren animals showed pronounced metabolic reallocations relative to kelp-margin animals, supporting energy production, redox balance, and membrane integrity. Carbohydrate metabolism was shifted towards increased glucose and gluconeogenesis with reduced alanine and glutamine as substrates for energy production. The pentose phosphate pathway fluxed towards energy production (supported by elevated sedoheptulose and mannoheptulose, and reduced glutamine and glutamate), rather than nucleotide synthesis and growth. Elevated amino acids (cysteine, cystine and lysine) reflected gonad protein catabolism, energy production and redox balance. Changes in lipid metabolism showed elevated monostearin from lipid breakdown, reduced heptanoic acid as energy substrate, and increased malonate supporting membrane integrity. This study reveals the specific metabolic adjustments purple sea urchins employ under food-limited conditions in barrens, diverting energy from reproduction to enhance long-term survival, potentially signaling challenges for kelp restoration.

## Introduction

Sea urchins are herbivores capable of overgrazing algal forests^1^. Urchin overgrazing results in a distinct ecosystem shift, as productive kelp bed habitat transitions to barren substrate^2^. The relationship between sea urchin grazing and kelp forest productivity can be stable for many years^3^ or change in relation to algal abundance, predator abundances, disease and storm disturbances^4^. Urchin barrens result in ecosystem productivity declines, accompanied by reduced food web complexity and species diversity. Associated economically important species may also decline, such as reduction in commercially harvested abalone species^5–7^, lobster^8^ and fish^9^. Certain urchin species demonstrate flexible foraging behavior, from passively consuming drifting algae to actively grazing on kelp that is attached to the reef^10^. When kelp subsidy (drift algae) supply is reduced and urchin energetic demand increases, urchins can shift behavior from drift foraging to active forest grazing, which can trigger barren formation. Urchins present in barren grounds with access to adequate drift algae can maintain normal metabolic activity and gonad mass^11,12^. However, even with food subsidies from external inputs^11^, many urchins in barrens likely endure extended periods of food scarcity^13^. Surprisingly, urchin barrens can last for decades as urchins have been reported to live up to 50-years on barren ground thanks to their ability to survive starvation in unknown ways, which are also accompanied by shifts to eating less nutritious microalgae and bacterial films and biota growing on open rock surfaces^2^.

In sea urchins, gonadal nutrient reserves can be harnessed by the organism to meet metabolic demands during periods of food scarcity, with low gonad indices seen as a hallmark of insufficient food supply^14^. Gonads can be depleted or restored within 6–8 weeks^12^ and are highly correlated with kelp cover (food availability) across the range^15^. Urchins are dioecious organisms and are of high commercial value due to their edible gonads, which are rich in high-quality protein and essential nutrients, such as amino acids, polysaccharides, lipids, vitamins and microminerals^16,17^. The urchin gonad serves as the primary organ for both reproduction and nutrient storage, with the percentage of reproductive and storage cells (nutritive phagocytes) varying based on position in the reproductive cycle^18^. Reproduction and gonadal development are affected by multiple factors, such as food, feeding patterns, quality of the feed, season^19^, water temperature, photoperiod^20^ and anthropogenic pollution^21^. Often, in barren areas, urchins have poor quality gonads, which coincides with food supply per number of sea urchins in the area^18,22^. Yet, recovery of reduced gonad mass quickly rebounds with feeding^13,23^. For example, a laboratory challenge utilizing purple urchins showed a decrease in metabolism in the absence of macroalgae, but restoration to normal metabolism within one week once access to macroalgae was returned^12^.

Many marine organisms facing food deprivation exhibit plastic changes in growth and body condition^24^, alongside less visible shifts in metabolism. Individuals can reduce or change metabolic demands by altering activity, behavior, physiology and metabolic pathways^12,25^. Sea urchins, for instance, can adjust size-specific metabolic rates^13^, however, the cellular mechanisms underlying these remain unclear. Understanding how food limitation modulates metabolism is crucial for predicting population responses to environmental changes^12^ especially for sea urchin populations which exert significant impacts in marine ecosystems. Under good nutritional conditions, urchins allocate energy to gonad production, with reserves stored in the body wall. When food is limited, reserves tend to be allocated to energy maintenance with gonadal growth paused^26^. Urchins and other marine organisms may also adapt to environmental changes by increasing their basal metabolic rate or redistributing energy for osmoregulation to support energy demands^27^. Moreover, starving animals can also lower metabolic costs by downregulating both the cellular demand for and the supply of adenosine triphosphate (ATP)^13^. Starvation typically involves glycogen degradation, the utilization of lipids to support physiological maintenance, and a switch to protein degradation once the lipids are depleted^28^. The physiological response of animals to limited food resources are controlled by interactions of cellular and molecular mechanisms, which drive overall metabolic changes^29^. Currently, we have a poor understanding of how sea urchins can maintain this downregulated state for periods of years when other marine invertebrates, such as abalone undergo complete starvation (no kelp provided) for up to 13 months before mortality occurs^30^.

These metabolic changes can be investigated using metabolomics, an omics-based approach aimed at the non-biased identification and quantification of metabolites in a biological system using highly selective and sensitive analytical techniques^31,32^. This discovery-driven evaluation of metabolite changes can provide insights into the regulation and response of organismal metabolism to nutritional and environmental changes^29,33^, as previously reflected in mussels^34^, abalone^35^ and shrimp^36^. Specifically, the use of gas chromatography-mass spectrometry (GC-MS), allows highly efficient and sensitive analyses with widely available libraries for metabolite identification. Additionally, GC-MS has the ability to separate and identify low-molecular-weight compounds (e.g., alcohols, hydroxyl, amino acids, sugars, fatty acids, sterols, and catecholamines), useful for nutritional studies^37,38^. The aim of this study is to use a metabolomics approach to derive a mechanistic understanding of the strategies sea urchins implement to survive starvation. In this study, we use GC-MS metabolomics to analyze gonad tissue of the purple sea urchin (*Strongylocentrotus purpuratus*) collected from sea urchin barrens comparing them with sea urchins collected from kelp margin habitats to better understand how they adapt to starvation conditions.

## Methods

### Urchin collection and dissection

Purple sea urchins were collected by commercial sea urchin divers from Shell Beach in Sea Ranch 38.42.13038 N, -123.11.30589 W), Sonoma County, northern California as part of a larger sea urchin removal study led by the Kashia Band of Pomo Indians. Sea urchins were collected from barrens habitats (*n* = 30) and along the kelp forest margin habitat (*n* = 27), and they were dissected at the UC Davis, Bodega Marine Lab on Sept. 9, 2024.

### Metabolomics analysis and data processing

Metabolite extraction was performed by adding a cold solvent mixture of acetonitrile: isopropanol: water (3:3:2, v/v/v) to a homogenized tissue aliquot of 25 mg sea urchin gonad followed by centrifugation and drying of the supernatant as per Fiehn^37^. For derivatization, 10 µL of methoxyamine hydrochloride in pyridine (20 mg/mL) was added to each sample and then shaken at 30 °C for 90 min, followed by the addition of 91 µL of a fatty acid methyl ester (FAMEs) and N-methyl-N-(trimethylsilyl) trifluoroacetamide (MSTFA) mixture, which was shaken for 30 min at 37 °C. Quality control (QC) samples were prepared by pooling gonad tissue from the biological samples. These pooled samples were treated as a biological sample and analyzed amongst the analytical batches to assess instrument repeatability and potential batch effects in the data. Additionally, a reference standard mixture, reagent and derivatization blanks and method blank extraction controls were included within the instrument batches and analyzed for QC purposes^37^. Samples were analyzed (0.5 µL injection volume) via an ALEX-CIS GCTOF mass spectrometry at the UC Davis Genome Centre Core Facilities, using a Rtx5Sil-MS column (Restek Corporation, USA, 30 m length x 0.25 mm internal diameter with 0.25 μm film made of 95% dimethyl/5% diphenyl polysiloxane) and gas flow rate of 1 mL/min helium flow. The GC oven was set to hold at 50 °C for 1 min, then ramp to 20 °C/min to 330 °C, then held for 5 min. The transfer line was set to 230 °C while the EI ion source was set to 250 °C. The mass spectrometer parameters collected data from 80 to 500 m/z at an acquisition rate of 17 spectra/sec^39^. Following the GC data acquisition, raw peak intensities were processed in ChromaTOF versus 2.32, generating the absolute spectra intensities. The peak intensities were further assessed by a filtering algorithm in the metabolomics BinBase database using protocols previously described by Fiehn^37^, and data were normalized by a normalization factor (mTIC), which was the sum of all peak heights for all identified metabolites (of only the known compounds). The acquired data set composed of the peak intensities was generated for a targeted mass inclusion list of metabolites with Fiehnlab BinBase database annotations^37^, database identifier [i.e., InChI key^40^, the compound annotation metadata (i.e., retention index, quantification mass, BinBase identifier, and mass spectrum), and PubChem and KEGG annotations^41,42^.

### Statistical analysis

The online webserver MetaboAnalyst (6.0) was used to detect metabolic differences in the GC-MS data^43^ between urchins collected from barrens and from kelp margins. Only features with metabolite identifications were considered for statistical analyses. Following generalized log (glog) transformation, univariate analyses (t-test) were performed to identify significantly different metabolites [*p* < 0.05, false discovery rate (FDR) ≤ 0.05]. Effect sizes (Cohen’s d-value) were calculated in Excel (Microsoft 365) as an indicator of practical significance, with the absolute difference between group means divided by the maximum standard deviation of the two groups determined and values of d > 0.8 considered a large effect^44^. Clustering as a heatmap and principal component analysis (PCA) were used to visualize metabolic variation and covariance among significant metabolites^45^. Body size was evaluated as a potential confounding factor using exploratory multivariate assessment; however, no size-related structuring or clustering was evident in the metabolomic profiles.

## Results

Purple sea urchins collected from barren habitats ranged from 48.6 to 78.8 mm in test diameter, with an average test diameter of 68.7 ± 8.3 mm (standard deviation) (*n* = 30). Sea urchins sampled at the kelp-bed edge ranged from 34.6 to 79.2 mm, with an average test diameter of 65.9 ± 8.4 mm (*n* = 27).

**Fig. 1:**
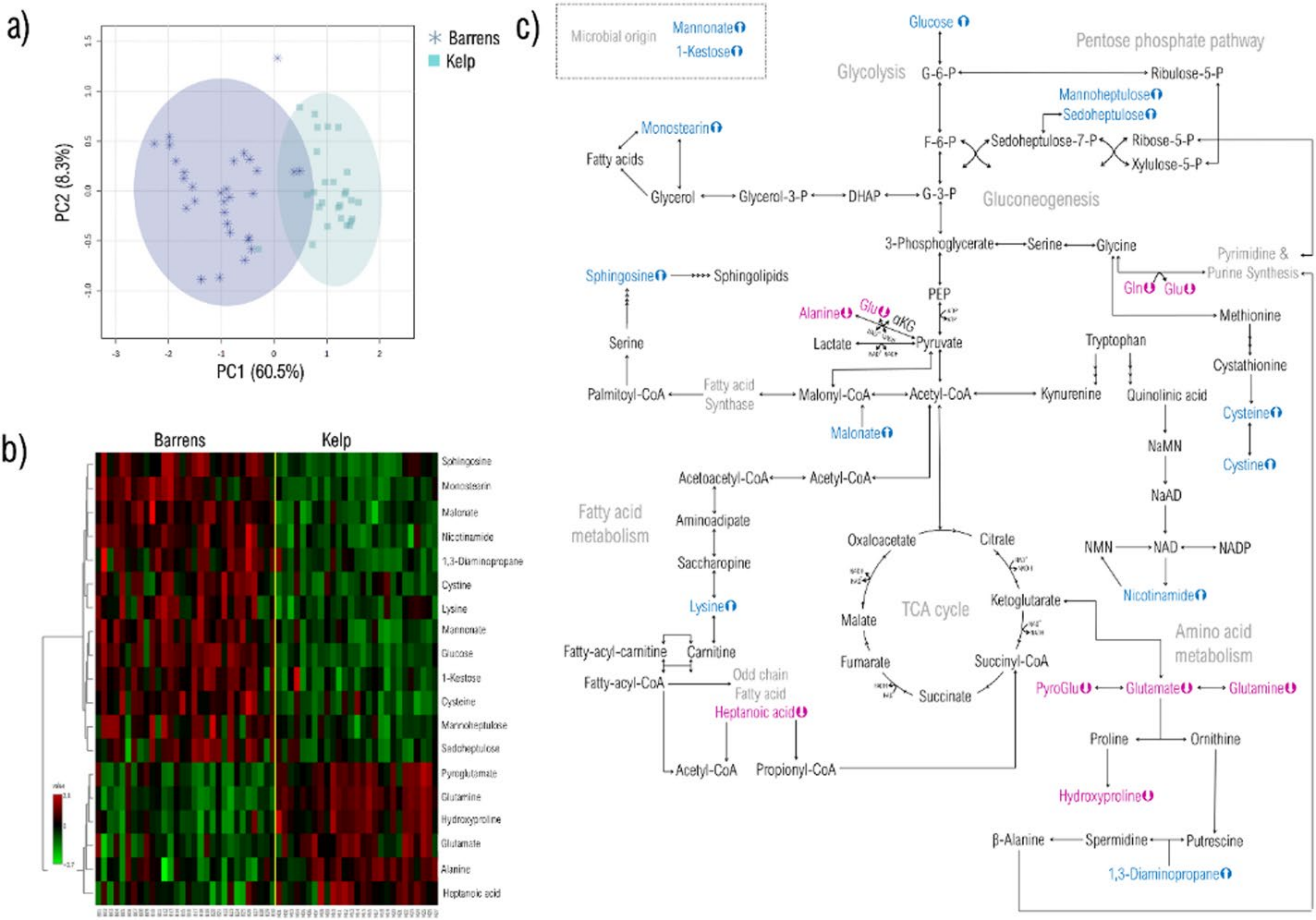
Overview of sea urchin gonad metabolomics results: (a) PCA score plots of metabolites of significance, indicating metabolite grouping between sea urchins collected from barren (*) and kelp (filled square) populations; (b) heatmap visualization of significant metabolites between sea urchins collected from barrens (left) and kelp (right) habitats (samples are in columns, and metabolites are in rows with colors varying from red to green shades in a numeric scale from 2.8 to − 3.7 to indicate metabolite abundance changes from high to low, respectively; c) metabolite map depicting identified metabolites as response of increased (blue ↑) or decreased (pink ↓) metabolite abundance in urchins collected from the barren population compared to the kelp habitat.

A total of 670 features (molecular signal defined by a unique mass-to-charge (m/z) ratio and retention time) were detected of which 126 were identified to the highest confidence levels pertaining to metabolite identification^46^. A total of 19 metabolites were found to significantly differ between the two experimental groups (Table 1). PCA analyses showed natural separation between the two groups with some overlap observed in the scores (Fig. 1a). Herein, the barren group of urchins showed the largest variation, with the first principal component (PC1) accounting for 60.5% of the variance between groups when considering significantly altered metabolites. The heatmap (Fig. 1b) provides an overview of the significant different metabolites (rows) grouping the metabolite abundances of urchins from barren (left) and kelp (right) populations. The metabolite response of urchins from the barren populations involves a range of metabolic pathways, which included glycolysis, the tricarboxylic acid (TCA) cycle, the pentose phosphate pathway, nucleotide metabolism, fatty acid synthesis and oxidation and amino acid metabolism (Fig. 1c). Most of the affected metabolites showed elevated metabolite levels, while alanine, heptanoic acid, glutamate, glutamine, pyroglutamate and hydroxyproline were reduced. Table 1 contains all discriminant information, i.e. the metabolite identifiers, associated metabolic pathways, effect size (d-value), t-test (*p*-value), FDR corrected *p*-value^47^ and the mean normalized metabolite abundance of both populations. The metabolite response focusing on significantly detected metabolites possessed by urchins from the barren population were plotted to schematically represent affected metabolic pathways.

**Table 1:** Metabolomics findings of gonad tissues obtained from sea urchins from barren populations.

Metabolite	PubChem ID	KEGG ID	Metabolic pathways	Effect size	T-test-*p*	FDR-*p*	Barren response	Mean (± STDEV) normalized abundance	
								Barren population	Kelp population
1,3-Diaminopropane	428	C00986	Polyamine metabolism	0.80	4.11E-06	3.99E-05	↑	2 843.93 ± 1 784	1 427.04 ± 741
1-Kestose	440080	C03661	Fructo-oligosaccharide / carbohydrate metabolism	0.81	3.00E-07	3.15E-06	↑	702.40 ± 335	329.56 ± 458
Alanine	5950	C00041	Amino acid metabolism / glucose-alanine cycle	1.18	2.24E-05	1.66E-04	↑	2 358 568.23 ± 1 073 404	3 821 642.07 ± 1 242 142
Cysteine	5862	C00097	Amino acid metabolism / glutathione synthesis / sulfur metabolism	0.80	9.45E-08	1.08E-06	↓	15 461.17 ± 12 956	5 064.44 ± 4 988
Cystine	595	C01420	Amino acid metabolism (oxidized dimer of cysteine)	0.89	5.31E-06	4.78E-05	↑	772.17 ± 564	268.07 ± 212
Glucose	64689	C00221	Carbohydrate metabolism / glycolysis / gluconeogenesis	1.45	3.28E-14	2.07E-12	↑	69 197.80 ± 39 985	11 391.52 ± 4 709
Glutamate	33032	C00025	Amino acid metabolism / nitrogen metabolism / TCA cycle intermediate	0.93	1.08E-04	6.45E-04	↑	127 575.57 ± 55 086	196 659.04 ± 7 4 445
Glutamine	5961	C00064	Amino acid metabolism / nitrogen shuttle / energy substrate	1.83	6.09E-16	7.67E-14	↓	129499.70 ± 79,027	531173.56 ± 219,464
Heptanoic acid	8094	C17714	Fatty acid metabolism / β-oxidation	0.80	4.42E-04	2.23E-03	↓	1 528.93 ± 763	2 535.70 ± 1 288
Hydroxyproline	5810	C01157	Collagen turnover / amino acid metabolism	1.23	1.93E-08	3.04E-07	↓	15 680.77 ± 6 726	30 480.30 ± 12 018
Lysine	5962	C00047	Amino acid metabolism / ketogenic amino acid	0.94	8.53E-06	7.16E-05	↓	2 697 946.23 ± 1 434 320	1 356 136.93 ± 521 136
Malonate	867	C00383	TCA cycle inhibitor / fatty acid synthesis intermediate	0.80	6.68E-12	1.68E-10	↑	1 446.57 ± 1 363	354.48 ± 217
Mannoheptulose	12600	C08236	Carbohydrate metabolism / glycolysis inhibitor	0.83	8.71E-05	5.49E-04	↑	1 401.40 ± 798	740.22 ± 248
Mannonate	3246006	C00514	Sugar acid metabolism / ascorbate biosynthesis precursor	1.13	9.63E-09	1.73E-07	↑	292.63 ± 165	105.59 ± 57
Monostearin	24699	D01947	Lipid metabolism / monoacylglycerol / fatty acid mobilization	1.11	4.86E-12	1.58E-10	↑	2 111.43 ± 1485	467.63 ± 215
Nicotinamide	936	C00153	NAD+/NADP+ metabolism / redox cofactor	1.19	5.02E-12	1.58E-10	↑	2 554.60 ± 1 388	896.52 ± 431
Pyroglutamate	7405	C01879	Glutathione metabolism / amino acid metabolism	1.34	2.81E-08	3.93E-07	↑	296 960.83 ± 120 369	540 125.22 ± 181 822
Sedoheptulose	5459879	C02076	Pentose phosphate pathway / carbohydrate metabolism	0.92	1.33E-04	7.59E-04	↓	713.23 ± 467	286.00 ± 160
Sphingosine	5280335	C00319	Sphingolipid metabolism / cell signaling / apoptosis regulation	0.80	9.06E-10	1.90E-08	↑	6 651.67 ± 7 776	629.07 ± 723

Metabolites are listed in alphabetical order, outlining the metabolite, metabolite identifiers as Pubchem and KEGG identification numbers, metabolic pathways, d-value, *p*-value, FDR *p*-value, the metabolite response from animals from the barren population as lower (↓) or higher (↑) metabolite abundance (compared to the kelp population), and mean normalized metabolite abundance of both populations.

## Discussion

GC-MS metabolomics was applied as a hypothesis-generating tool, to investigate the differences in the gonad metabolite profiles between purple sea urchins collected from kelp barren and adjacent kelp margin populations. The resulting metabolite patterns support the utilization of carbohydrate, protein and lipid reserves as primary mechanisms sustaining urchin survival. The utilization of glycogen, lipids, proteins and fatty acids have been previously reported in the oyster, *Crassostrea gigas*,^28^ and clams, (*Ruditapes decussatus* and *Venerupis pullastra*)^48^ in response to starvation and is now reported for the first time in the sea urchin, *Strongylocentrotus purpuratus*. Metabolomics studies focusing on starvation in echinoderms remain limited, but studies on the crown-of-thorn starfish have documented starvation-induced changes at the gene expression level, linked to glycolysis and the citrate cycle^49^ and at the microbiome level, suggesting adaptions in microbial networks during starvation^50^, supporting similar responses of the current investigation, albeit at different levels of biological organization.

Carbohydrates have diverse purposes, serving as structural and energy storage molecules^51^. In this study, elevated glucose was detected in the gonad tissue of *S. purpuratus* from the barren population, likely as a stress coping mechanism related to nutrient scarcity. Glucose is used to drive glycolysis and subsequent energy production^34^. Typically, during periods of fasting, circulating glucose will decrease, necessitating the breakdown of glycogen to produce glucose. Gluconeogenesis also allows the synthesis of glucose from other metabolites, such as amino- or fatty acids^52^. Additionally, trehalose can be hydrolyzed to glucose. While not detected in the present study, increased trehalase activity and transcript levels have been reported in *S. purpuratus* larvae under low food conditions^51^ suggesting the involvement of trehalose-associated carbohydrate pathways. Trehalose also functions as a stress-protective molecule involved in maintaining membrane integrity in bacteria, yeasts^53^ and insects^54^. Collectively, these findings highlight the role of carbohydrate metabolic pathways in sea urchins under nutrient-limited conditions, with evidence from other organisms indicating their involvement in regulating stress responses.

Apart from the aforementioned changes to carbohydrate metabolism, protein reserves are also typically used as alternative energy substrates during times of stress and nutrient deprivation. Higher amounts of free amino acids are often reported in aquatic invertebrates subjected to stress, as a result of the intracellular production of amino acids from protein breakdown to support energy production via glucogenic pathways^29^. In the sea urchin, *Paracentrotus lividus*, exposed to starvation, lower amino acid excretion and poor gonad growth was reported to be associated with protein breakdown^14^. In this investigation, elevated concentrations of various amino acids (cystine, cysteine, lysine, diaminopropane) were detected in the gonad tissue of the barren habitat sea urchins, supporting this phenomenon. Previous reports indicate, that sea urchins in general, have a high cysteine content, which serves various important roles including: (1) reinforcing intermolecular disulphide bonds, for maintaining protein structure^55^, (2) binding sites for heavy metals, supporting detoxification processes^56^, (3) regulating osmotic pressure by enhancing taurine synthesis^27^ and (4) maintaining the redox state and supports antioxidant functions^57–59^. Higher levels of cysteine in the urchins under study may therefore contribute to oxidative stress management (pertaining to redox reactions and antioxidant functions), as imbalances between reactive oxygen species production and antioxidant defenses can occur under stress conditions, including starvation^60^. Lysine is a α-ketogenic amino acid, primarily degraded via the saccharopine pathway to produce acetyl-coenzyme (Co) A, which in turn is used as an energy substrate for the TCA cycle. During periods of starvation, ketogenic amino acids along with fatty acids produce acetyl-CoA to drive ketogenesis and the regeneration of nicotinamide adenine dinucleotide (NAD^+^)^61^. However, during stress, marine organisms have been shown to rely more heavily on acetyl-CoA derived from lipid catabolism, with a reduced dependence on lysine degradation^62^. This shift explains the elevated lysine levels observed in the barren urchin group in our study. In addition to its role in energy metabolism, lysine is essential for protein biosynthesis^63^ and is incorporated as lysine residues during translation^64^. In sea cucumbers, experiencing heat stress, lysine acetylation was outlined as an important post-translational modification in signaling pathways of diverse stress responses^65^. Lysine acetylation supports gene regulation, deoxyribonucleic acid protein interactions and protein stability, while also ensuring metabolic adaptive mechanisms by altering enzyme activities as a stress response^66^. The increased lysine detected in the sea urchins in the present study supports the metabolic shift towards protein catabolism and an adaptive stress response, rather than protein synthesis. The elevated concentrations of nicotinamide, the precursor to NAD^+^, further supports the imbalance in redox state and energy metabolism of the barren habitat sea urchin population in this study^67–69^.

Furthermore, various concentrations of those amino acids directly associated with immediate energy production, including the glucogenic amino acids; alanine, glutamate and glutamine, along with pyroglutamate and hydroxyproline, were seen to be reduced in the sea urchin gonads from the barren habitat population in this study. Glucogenic amino acids are typically converted to alanine via the transamination of pyruvate. Alanine can also transfer its amino group to α-ketoglutarate through a transamination reaction, producing glutamate (which can subsequently be aminated to form glutamine) and pyruvate^70^. During periods of starvation, both alanine and glutamine serve as key gluconeogenic amino acids, and are ultimately converted to oxaloacetate to initiate gluconeogenesis^71^. Supporting this, analyses of the coelomic fluid of black sea urchins (*Mesocentrotus nudus*), showed alanine as an alternative energy substate to food restriction^72^. Glutamate, via conversion to α-ketoglutarate, a key TCA cycle intermediate, serves an anaplerotic role in energy metabolism^73^. Glutamate can also be deaminated via glutamate dehydrogenase to regenerate α-ketoglutarate and release ammonia, a mechanism well-document in vertebrates for nitrogen and anaplerotic balance^74^. However, this pathway is poorly characterized in invertebrates where experimental validation of metabolic fluxes is largely lacking^75^. Pyroglutamate, a glutamate analogue and precursor, helps maintain intracellular glutamate concentrations and has also been linked to various osmoprotective roles^76^. Thus, the reduced levels of both glutamate and pyroglutamate observed in barren habitat sea urchins from this study likely reflects their use as energy substrates, coinciding with an increased need for NADH production from alternative sources under conditions of nutrient scarcity. Glutamate is also a precursor in glutathione biosynthesis, which plays a crucial role in antioxidant defense and redox balance^77^, while pyroglutamate is an intermediate of glutathione degradation^76^. These findings support the hypothesis that we are observing elevated oxidative stress in barren urchins. Hydroxyproline (detected in comparatively reduced amounts in the barren urchins under study) and proline contribute to collagen synthesis and tissue repair, regulation of polyamine production, enhancement of protein synthesis, and scavenging of free radicals^78^. A reduction in proline (precursor to hydroxyproline) was reported in *Diporeia* (amphipod) following starvation, serving as a major source of free amino acids for energy production^33^. Hence, the reduced levels of hydroxyproline in sea urchins from the barren habitat in this study also supports the hypothesis of increased catabolism of these amino acids to drive energy production and defenses against cellular oxidative stress under nutrient-limited conditions.

Along with the glycolysis pathway, the pentose phosphate pathway (PPP) also contributes to carbohydrate and amino acid catabolism^79^. In this study, higher levels of sedoheptulose were found in the barren habitat sea urchin population, formed by the dephosphorylation of sedoheptulose-7-phosphate, which is an intermediate of the PPP^80^. Under normal conditions, this pathway ensures precursors for the synthesis of nucleotides, amino acids and vitamins for growth and replication^81^, while also regulating redox flux reactions to prevent oxidative stress^82^. A similar increased response of the PPP was found in the sea cucumber, *Apostichopus japonicus*, in response to estivation (when feeding stops), suggesting an increased carbon flow into the PPP, as an antioxidant mechanism^77^. Alongside sedoheptulose, its isomer mannoheptulose was also detected as increased in the barren habitat sea urchin population. The seven-carbon (C7) sugars, mannoheptulose and sedoheptulose, are widely distributed in algae^83^, and are reported to regulate carbon flux^84^. Considering that urchins from the barren population, likely had access to less algae, the increased state of these metabolites potentially suggests either sources from diatoms, endogenous synthesis or mobilization from internal stores (rather than dietary intake) as a potential compensatory mechanism to sustain the PPP, to sustain the redox balance. The PPP also supports de novo nucleotide synthesis by contributing to the synthesis of glutamate and glutamine^85^, key amino acids that serve as primary nitrogen donors in purine and pyrimidine biosynthesis^86,87^. Thus, lower levels of these metabolites in the starved urchin group would impair nucleotide synthesis, potentially leading to increased reliance on salvage pathways to maintain nucleotide pools. Further confirming this is the elevated amounts of 1,3-diaminopropane, a naturally occurring polyamine, that functions as a byproduct of polyamine degradation^88^, and contributes to the production of metabolites such as beta (β)-alanine, which is involved in pyrimidine metabolism^89^. Collectively, polyamines contribute to synthesis, functioning, maintenance, and stability of nucleic acids, and support cellular processes related to transcription, translation, signaling, and post-translational modifications^90^. Considering this, the elevated concentrations of these metabolites (sedoheptulose, mannoheptulose and 1.3-diaminopropane) are likely due to reduced growth and nucleotide synthesis due to nutrient deprivation, and a redirection of this pathway towards energy generation and preventing oxidative stress.

Fatty acid metabolism via β-oxidation also supports energy production and has been highlighted as a significant functional pathway during gonadal development and growth in *S. intermedius*^16^. In the present study on *S. purpuratus*, monostearin was detected in elevated concentrations in the sea urchins’ gonad tissue from the barren habitat population, indicating an increase in fat catabolism, as the sea urchins rely on not only stored protein, but also stored lipids to sustain energy and essential physiological functions, as is the case in other marine organisms during periods of hibernation^84^. A decrease in the fatty acid metabolite heptanoic acid was also observed, indicating an increased use of fatty acids as an alternative energy source, via β-oxidation to acetyl-CoA and propionyl-CoA^85^.This use of fatty acids is further reinforced by elevated malonate levels in barren urchins, as malonate is converted to malonyl-CoA via the ATP-dependent enzyme acetyl-CoA carboxylase, the primary step in fatty acid synthesis^91^. Together these changes indicate that while fatty acid breakdown is upregulated to meet energy demands, fat synthesis via malonyl-CoA is likely reduced in gonadal tissue under starvation.

Next, elevated levels of sphingosine were observed in the barren urchin population. Sphingosine, considered the backbone of sphingolipids, is a key component of cell membranes, and involved in cell signaling, membrane structure, and are associated with cell-growth arrest and the induction of cell death^92,93^. Similar functions have been reported in *Strongylocentrotus intermedius* under low-salinity stress, where sphingosine-1-phosphate metabolism, was implicated in preventing apoptosis^27^. Likewise estivating sea cucumbers, showed increased sphingolipid derivatives which served a protective function to inhibit apoptosis during a low metabolic state^77^. In contrast, down-regulation of glycerophospholipid and sphingolipid metabolism, was reported in starved amphipods (*Diporeia*)^33^ highlighting species-specific metabolic strategies for coping with nutrient limitations. Considering this, the increased sphingosine levels observed in the barren urchins in the present study is likely an adaptive mechanism to low-nutrient conditions providing a means to instantiate apoptosis if starvation stress is prolonged.

Two metabolites that were observed to be elevated in barren urchins from our analysis, namely 1-kestose and mannonate, are likely of microbial origin. The metabolite, 1-kestose is considered a fructo-oligosaccharide (i.e., a prebiotic compound), that cannot be hydrolyzed by the gastrointestinal track and is fermented by hydrolytic microbes in the gut^94,95^. The second loop of the gut contains the highest amount of carbohydrate digesting bacteria^96^. When used as a prebiotic, 1-kestose has been shown to increase the population of beneficial microorganisms in vitro and in vivo^97^. Literature suggests that 1-kestose is present in a wide range of plants, which synthesize inulin as carbohydrate reserve, serving as an energy storage molecule, from sucrose with 1-kestose found as the first product in the formation of inulin^98^. Additionally in bacteria, hexuronate metabolism breaks down sugar acids to convert mannonate to 2-keto-3-deoxygluconate to support energy production where glycolysis is not utilized^99^. No primary literature evidence to support the presence of 1-kestose or mannonate in urchins or in their macroalgae diet could be found, highlighting this as an area of interest for future research. We hypothesize that the concentration of these metabolites may be driven by a change in the microbiome of barren urchins. Considering that urchins from the barren populations have a reduced available diet, these compounds are likely synthesized to support gut function and promote the health of the host in the absence of normal food quality and quantity and associated microbiota.

In general, animals are well-adapted to mobilize metabolic responses and body constituents to survive periods of food scarcity^100^. During periods of starvation, animals adaptively activate endogenous metabolic processes to meet their energy needs^101^. For example, long-term starvation induces metabolic depression^102^, which compromises other fitness-related functions and lowers the animals capacity to cope with other stressors^28^, and hence metabolic adaptation is required to ensure survival. Sea urchins are particularly adept at surviving starvation^13^ and here we investigate the metabolic pathways that are activated to facilitate survival during starvation conditions. We elucidated the metabolic adaptations of purple sea urchins within barrens habitats that utilize their gonad tissue for survival rather than reproduction. Through this line of inquiry, metabolomics can be used as a heuristic tool to better understand how urchin barrens are maintained, generating novel insights into the physiological mechanisms utilized by urchins that contributes to the persistence of barrens and suppression of kelp forest recovery^32^ seen in northern California^5,6^ and worldwide^3^. The metabolomics analyses herein newly report a multi-layered metabolic response in the gonad tissue of the purple sea urchins collected from nutrient-poor barren grounds. In essence, the results provide insight into the metabolic response to food deprivation, demonstrating that the gonad tissue of animals from barren grounds are utilized to support survival. The results show the breakdown of protein, lipid and carbohydrate reserves supporting internal resource mobilization with affected metabolites linked to polyamine-; carbohydrate-; amino acid-; fatty acid-; sugar-; redox-; and sphingolipid metabolism. While shifts in carbohydrate, amino acid and lipid metabolism indicate altered substrate utilization under nutrient-limited conditions, direct impacts on cellular energy status cannot be inferred, as changes in adenylic nucleotides, phosphagens, and cellular energy charge were not detected in this study. Future work could examine the mechanisms that contribute to how long sea urchins can persist in long-lived urchin barrens. The ability of purple sea urchins to metabolically adapt to a low food environment as demonstrated by reduced reproductive output, and poor gonad quality can result in the loss of sea urchin roe as a valuable fishery resource. Further, resilience to starvation as demonstrated by the metabolic adaptations of purple sea urchins highlighted in this study suggests that these sea urchins will not simply die off following the collapse of the kelp forest, posing a significant challenge for kelp forest restoration. Finally, this study demonstrates the utility of metabolomics as a tool to assess organismal condition and detect environmentally driven physiological shifts, with applications in resource management and restoration.

## Data Availability

The dataset generated and analyzed during the current study are available from the corresponding author on reasonable request.
